# Ultra-Porous Nanoparticle Networks: A Biomimetic Coating Morphology for Enhanced Cellular Response and Infiltration

**DOI:** 10.1038/srep24305

**Published:** 2016-04-14

**Authors:** Noushin Nasiri, Anthony Ceramidas, Shayanti Mukherjee, Anitha Panneerselvan, David R. Nisbet, Antonio Tricoli

**Affiliations:** 1 Nanotechnology Research Laboratory, Research School of Engineering, Australian National University, Canberra 2601, Australia; 2 Laboratory of Advanced Biomaterials, Research School of Engineering, Australian National University, Canberra 2601, Australia

## Abstract

Orthopedic treatments are amongst the most common cause of surgery and are responsible for a large share of global healthcare expenditures. Engineering materials that can hasten bone integration will improve the quality of life of millions of patients per year and reduce associated medical costs. Here, we present a novel hierarchical biomimetic coating that mimics the inorganic constituent of mammalian bones with the aim of improving osseointegration of metallic implants. We exploit the thermally-driven self-organization of metastable core-shell nanoparticles during their aerosol self-assembly to rapidly fabricate robust, ultra-porous nanoparticle networks (UNN) of crystalline hydroxyapatite (HAp). Comparative analysis of the response of osteoblast cells to the ultra-porous nanostructured HAp surfaces and to the spin coated HAp surfaces revealed superior osseointegrative properties of the UNN coatings with significant cell and filopodia infiltration. This flexible synthesis approach for the engineering of UNN HAp coatings on titanium implants provides a platform technology to study the bone-implant interface for improved osseointegration and osteoconduction.

The rapid rise of nanofabrication technologies is enabling the engineering of hierarchical materials and surfaces that mimic the complex morphology and composition of biological tissue^1^,^2^. This is critical to bone tissue engineering where the interaction between the synthetic implant and bone is determined by the implant’s surface properties. Bone injuries account for a large proportion of surgeries and healthcare expenditures^2^,^3^. These procedures often require the permanent implantation of synthetic structures such as prostheses and grafts in the bone. The success of these implants is determined by their osseointegration with the surrounding bone tissue. This long-term integration requires materials stability and careful engineering of biomimetic interfaces. This is challenging as mammalian bone is a composite material composed of hierarchically assembled mineralised collagen together with a small portion of non-collagenous protein and lipids. This assembly of mineralised collagen fibrils can lead to varied bone structure and morphologies depending on its location and function. The most biologically active section of mammalian bones, namely the trabecular region, features a honey-comb type spatial arrangement of mineralised collagen fibrils^4^,^5^ with a porosity range of 30–95%^6^,^7^.

Currently, much effort is focused on the nanofabrication of enhanced HAp coatings that may ultimately improve the integration of orthopaedic implants. Synthesis of nanostructured HAp coatings is currently pursued by numerous methods including plasma spraying^8^, sol-gel^9^,^10^ and magnetron sputtering^11^. Plasma spraying is the gold standard for the commercial production of implant coatings. Notwithstanding its numerous merits, this approach results in dense films with limited chemical homogeneity and mechanical properties^12^. These two-dimensional plasma-sprayed interfaces do not adequately mimic the highly porous three-dimensional structure of bone tissue and may result in shorter *in vivo* implant lifetime compared with a coating with superior biomimicry^13^. In other studies^10^,^14^, porous HAp coatings (15.5–75% porosity) were fabricated using micro-porous biphasic calcium phosphate (BCP) granules mixed with organic pore makers^14^ and sol-gel^10^. The resulting calcium phosphate was amorphous and calcination at 600 °C^10^ to 1050 °C^14^ was required for crystallization.

Thermo-15 and electrophoretically-driven^16^ deposition of nanoparticle aerosols has been, recently, demonstrated as a flexible tool for the synthesis of three-dimensional morphologies^17^ with excellent optoelectronic^18^ and chemical properties^19^. This gas-phase approach shares some of the advantages of plasma-spraying such as scalability and high deposition rates while enabling fabrication of up to 98% porous morphologies^20^ and more accurate control of the key structural features^21^. However, previous attempts to produce crystalline HAp aerosols by high-throughput spray flames have led to amorphous compounds^22^,^23^,^24^. Furthermore, these ultra-porous nanoparticle coatings are usually characterized by a fragile structure that is easily destroyed by capillary forces^18^, and are not suitable for biological applications. Enhancing their mechanical stability for operation in liquid environments has been only partially successful^25^.

Here, we demonstrate the synthesis of a three-dimensional nanocrystalline HAp morphology that mimics the high porosity and micro-nano structural hierarchy of the trabecular bone region. Ultra-porous nanoparticle networks of crystalline HAp are fabricated in one-step by rapid self-assembly of meta-stable core-shell nanoparticle aerosols and *in-situ* thermally-induced self-organization. This results in ultra-porous and micro-rough films composed of strongly sintered nanostructures that withstand capillary forces and handling. Up to five to ten times denser morphologies are obtained as a comparative structure by classical spin-coating of the same flame-made nanoparticles. The performance of this synthetic UNN morphology is assessed by primary osteoblast cultures revealing unique cellular response and infiltration. We believe that this rapid and scalable synthesis route offers a powerful platform for the large-scale and low-cost fabrication of ultra-porous inorganic coatings with application extending from regenerative medicine to implantable devices.

## Results and Discussion

### Flame Synthesis of Core-Shell Nanoparticle

**Figure 1:**
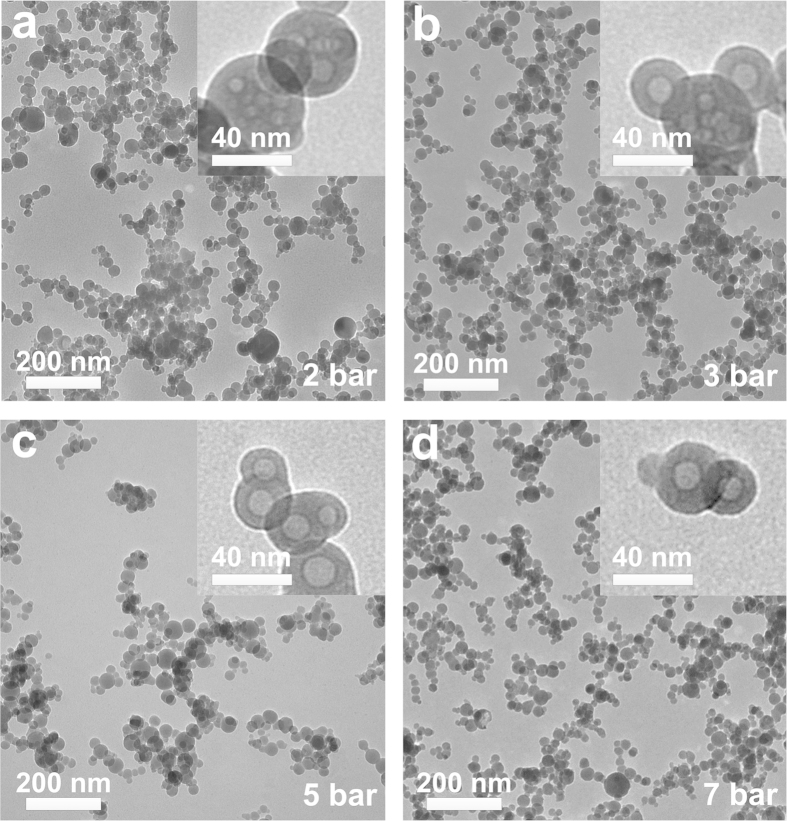
Representative transmission electron microscopy images of the flame-made HAp nanoparticle aerosols at an atomization pressure of (**a**) 2 bar, (**b**) 3 bar, (**c**) 5 bar and (**d**) 7 bar.

Highly concentrated aerosols of hydroxyapatite (HAp) nanoparticles were produced by combustion of calcium naphthenate in tributyl phosphate solutions (Figure S1). Previous attempts to produce crystalline hydroxyapatite nanoparticles by highly turbulent and scalable spray flames^26^ have usually resulted in nearly completely amorphous Ca/P compounds^24^. To overcome this limitation, here, a custom-built atomizer design (Figure S1) has been utilized that enables higher atomization pressure than previously investigated (≈1.5 bar)^24^. Figure 1 shows the effect of the atomization pressure on the nanoparticle size and morphology. The atomization pressure was increased from 2 to 9 bar while maintaining a constant flow rate by radially decreasing the annular cross-section of the atomization gas (O_2_) outlet.

**Figure 2:**
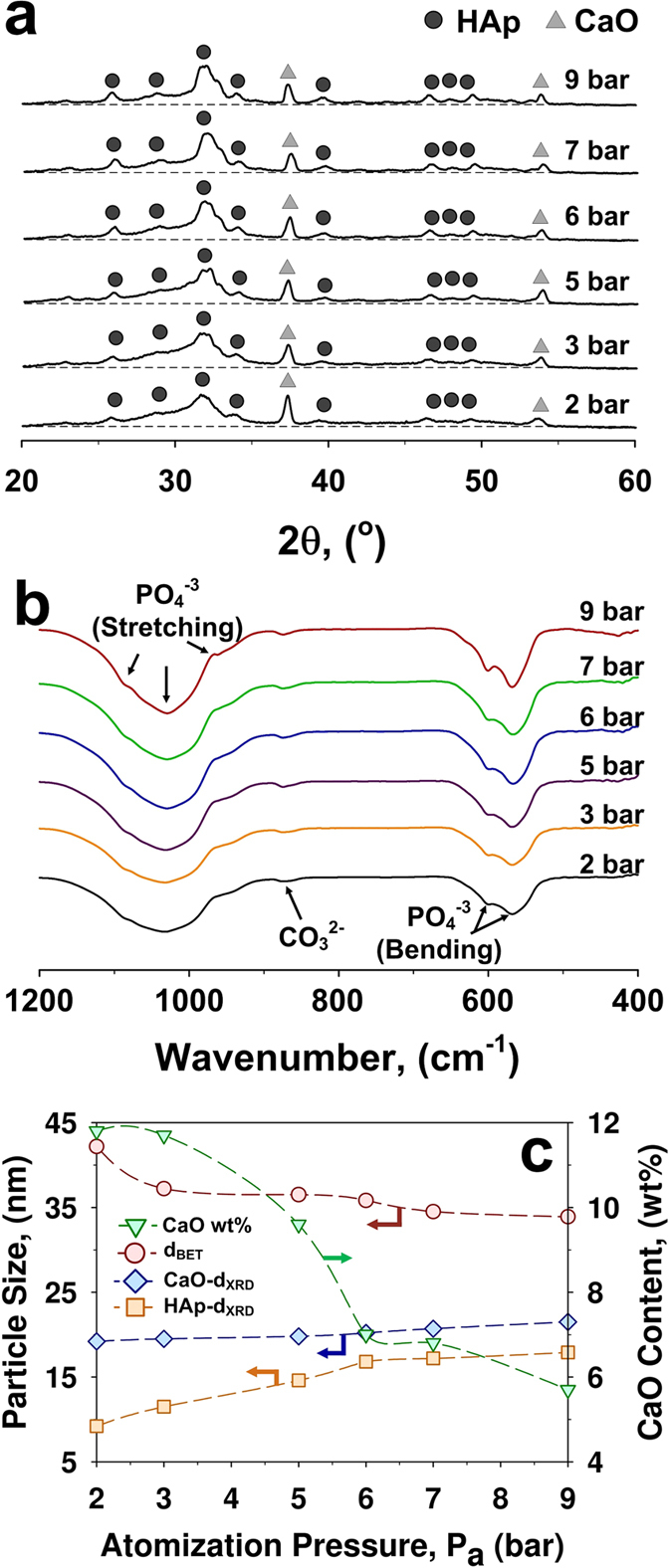
Composition of the as-prepared HAp nanoparticles as a function of the atomization pressure by (**a**) X-ray diffraction and (**b**) Fourier transform infrared spectroscopy. The (**c**) particle size (d_BET_), crystal size (d_XRD_) and calcium oxide content of HAp nanoparticles collected from filter produced at atomization pressure from 2 to 9 bar.

For all conditions, transmission electrons microscopy (TEM) analysis of the nanoparticle aerosols revealed a fraction of spherical core-shell nanoparticles (Fig. 1a–d, insets). These particles were characterized by bright cores and dark shells (Fig. 1a–d, Figure S1) indicating a phase segregation. X-ray diffraction (XRD) revealed a crystalline structural (Fig. 2a) composed of two phases matching the peaks of hydroxyapatite (Ca_5_(PO_4_)_3_OH) and calcium oxide (CaO). At low atomization pressure (2 bar), some trace amounts of amorphous materials and/or very small crystals (<3 nm) were indicated by an amorphous hump centred at a 2 θ of 31° (Fig. 2a). The intensity of the latter decreased with increasing atomization pressure and above 5 bars highly crystalline powders were obtained. Similarly, the content of the calcium oxide impurities decreased significantly from 12% to 6% with increasing atomization pressure from 2 to 9 bar (Fig. 2a,c). These results were confirmed by FTIR analysis (Fig. 2b). Bands commonly assigned to phosphates were observable in all samples at ~ 563, 604, 962, 1029 and 1087 cm^−1^ 27. The bands at 962, 1029 and 1087 cm^−1^ are attributed to the characteristic symmetric stretching of the phosphate (PO_4_^−3^) in HAp while those at 563 and 604 cm^−1^ are attributed to its bending mode^28^. A small hump centred around a wavenumber of ~875 cm^−1^ suggests partial replacement of PO_4_^−3^ in HAp lattice by carbonate^29^. The incorporation of carbonate is common during the formation of biological apatite^30^.

Following the XRD phase composition, the nanoparticles’ dark shells (Fig. 1a–d, Figure S1) are attributed to the higher mass density of the CaO phase while the bright cores are associated with the hydroxyapatite phase in line with previous reports on segregated multi-components nanoparticles^31^,^32^. This is in good agreement with the high fraction of single-core structures observed at high atomization pressures and higher fraction of multi-core particles at low atomization pressures (Fig. 1a–d, insets). In fact, the HAp crystal size increased from 9 nm to 17.5 nm and the calcium oxide crystal size increased from 19 nm to 21.5 nm with increasing atomization pressure from 2 to 9 bar (Fig. 2c). The high amount of crystalline HAp, obtained here, is attributed to the high partial water pressure^33^ obtained by oxidation of the precursor solution. Its segregation in the particle core is tentatively attributed to the rapid heating and high temperatures experienced by the particles during their residence time in the flame that may lead to dehydroxylation of their surface. Higher atomization pressures are expected to result in smaller droplets and a shorter high temperature residence time. This could decrease surface dehydroxylation and promote the formation of larger crystalline nanoparticle cores. Increasing the moderate temperature residence time (e.g. with an hot wall reactor) of the optimally synthesized core-shell nanoparticles at an atomization pressure of 7 bar is expected to eventually lead to fully crystalline single-phase HAp nanoparticles. This is in good agreement with the TEM analysis (Fig. 1d, inset) showing an increase in HAp core size with increasing atomization pressure from 2 to 7 bar. As a result, a pressure of 7 bar was chosen as the optimal atomization condition as it led to 94 wt% of crystalline HAp phase and warranted stable spray flame conditions.

**Figure 3:**
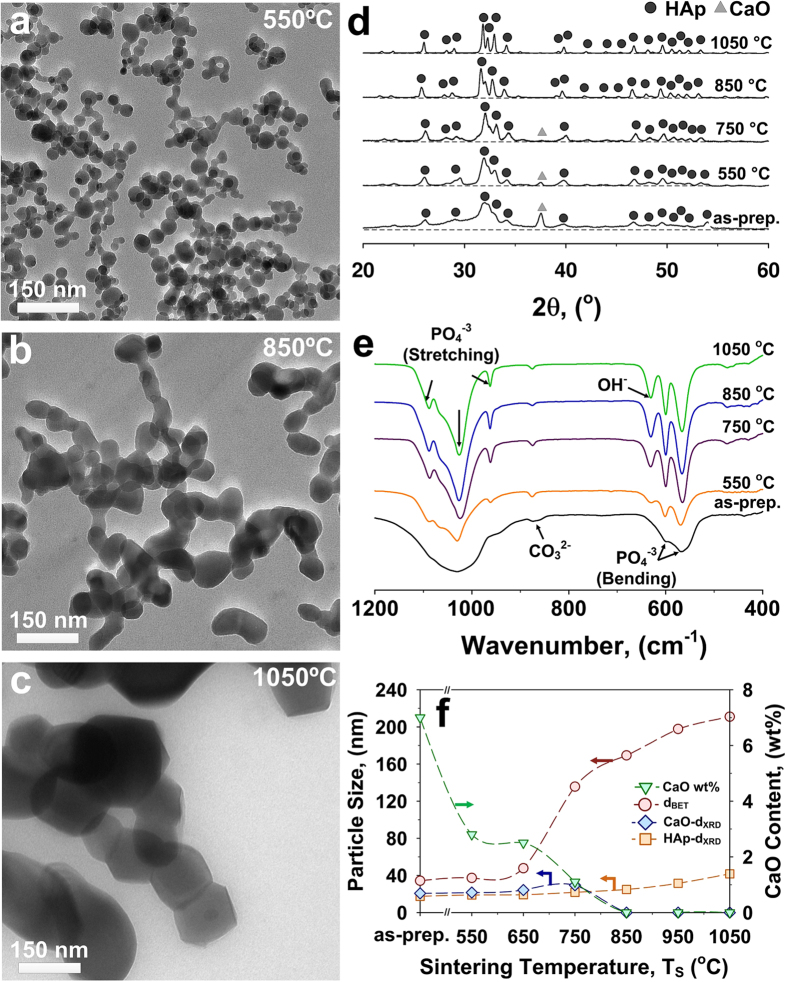
Representative TEM images of flame-made HAp nanoparticles upon sintering for 1 h at (**a**) 550 °C, (**b**) 850 °C and (**c**) 1050 °C. The (**d**) XRD patterns, (**e**) FTIR spectra, (**f**) particle size (d_BET_), crystal size (d_XRD_) and CaO content of HAp nanoparticles as a function of the sintering temperature.

The synthesis mechanism of these core-shell nanoparticles is further analyzed below along their process parameters and thermodynamic stability. The sintering temperature had a significant impact on the particle morphology and composition (Fig. 3a–c). Already at 550 °C, formation of hard agglomerates was observed and the as-prepared core-shell morphology was replaced by a homogeneous composition (Fig. 3a–c). This is in contrast with the high thermal stability of crystalline HAp and suggests that the as-prepared nanoparticles are meta-stable nanocomposites captured due to the strong quenching in the flame reactors. In fact, the particle diameter (d_BET_) increased from 35 to 210 nm with increasing sintering temperature from as-prepared to 1050 °C (Fig. 3c,f, red circles). Simultaneously, the HAp and calcium oxide crystal sizes increased from 17 and 20 nm to 22 and 30 nm, respectively, with increasing sintering temperature from as-prepared to 750 °C (Fig. 3f). This also led to a rapid drop in the CaO content from 7 wt% to less than 1 wt% (Fig. 3f, green triangles). Sintering temperatures above 850 °C led to a pure HAp phase with no detectable trace of calcium oxide and other impurities (Fig. 3d).

These nanoparticle aerosols were further investigated by DSC and FTIR analysis. The former (Figure S2) indicates that no amorphous to crystalline phase transformation and decomposition occurs up to 1000 °C. This is in very good agreement with previous studies^23^,^34^ and the very low content of calcium oxide impurities (≤6 wt%) of the as-prepared samples. The small nicks observed below 700 °C and close to 900 °C are within instrument accuracy. Figure 3e shows the FTIR analysis of the HAp particles as a function of the sintering temperatures. Compared to the as-prepared particles (Fig. 2b), the sintered ones showed characteristics of larger HAp crystals with well distinguishable FTIR peaks. A major difference notable in the sintered samples was the presence of an OH band at 630 cm^−1^. In contrast, the FTIR spectra of the (as-prepared) particles containing CaO did not have this band. This is commonly attributed to the stretching vibration mode of lattice OH^27^. Its post-sintering appearance is attributed to the transformation of the calcium oxide shell (CaO) into stoichiometric HAp (Ca_5_(PO_4_)_3_OH), which is in good agreement with the XRD analysis (Fig. 3d).

### Rapid Nanofabrication of Biomimetic and Robust UNN

**Figure 4:**
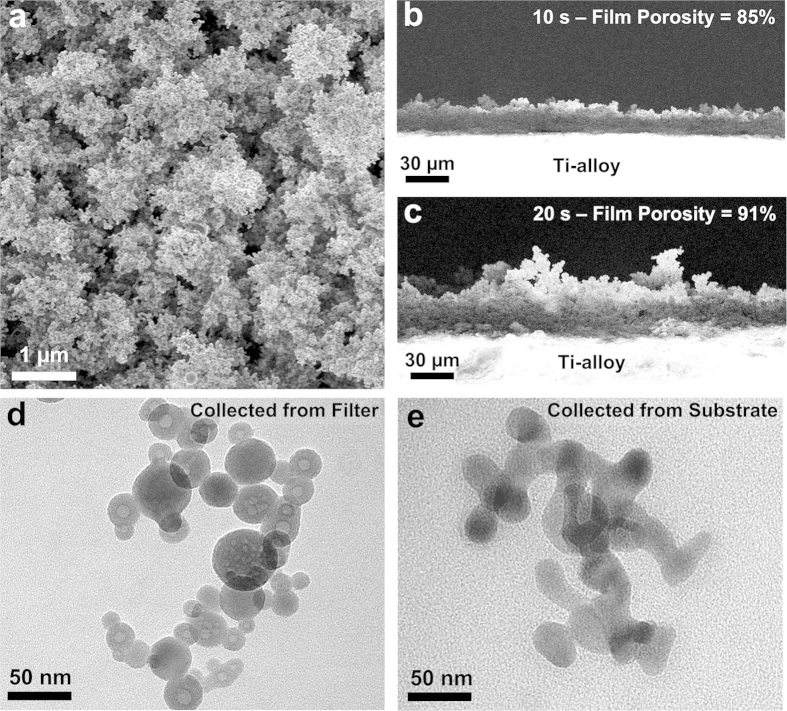
(**a**) Representative SEM images of an ultra-porous HAp nanoparticle network (UNN) self-assembled for 10 s on the surface of a Ti-alloy substrate. Cross-section SEM analyses of the (**b**,**c**) UNN coatings obtained at 10 and 20 s aerosol-deposition time. The cross-section SEM images in panel b and c are at the same scale. TEM images of the particles collected from (**d**) the filter and (**e**) removed from the substrates after 5 s aerosol-deposition.

Ultra-porous nanoparticle networks were rapidly (<30 s) fabricated by hierarchical self-assembly of these aerosols on Ti-alloys, commercially utilized for bone implants. The substrates were orthogonally aligned to the spray flame (Figure S1) and at 6 cm height above the burner head resulting in a surface temperature of 1000 ± 10 °C. This temperature is lower than that commonly utilized for commercial plasma spraying coating (>1200 °C)^35^,^36^ and did not have any detectable effect on the substrate. Figure 4a shows a typical UNN film obtained by 10 s exposure of the substrate to the hot nanoparticle aerosols. The resulting film had a three-dimensional micro- and nano-scale surface roughness (Fig. 4a) characterized by large micro-scale pores surrounded by dense agglomerated regions. Analysis of the film cross-sections (Fig. 4b) revealed a homogeneous morphology over several hundred micrometres with a thickness of 19 μm and variations in the order of ±2 μm. The substrate-film interface was continuous with no visible cracks and delamination suggesting a robust bond between the film and the Ti-alloy. An initial analysis of the bonding strength of the HAp/Ti interface was performed by 10 cycles abrasion tests with a Taber abrasion instrument revealing a promising mechanical stability. Further studies are required to provide a full characterization of the structural properties of these coatings. Figure S3 shows SEM micrographs of the HAp coatings before and after 10 cycles abrasion indicating minimal morphological changes. Furthermore, in contrast to previous studies on UNN, the HAp films survived the capillary forces during immersion in simulated body fluid^18^.

Increasing the deposition time from 10 to 20 s, increased the film thickness from 19 to 36 μm (Fig. 4b,c). This results in a very high film growth rate of 1.8 μm s^−1^. Constant, but significantly lower film growth rates of 7 nm s^−1^ have been reported also for deposition of flame-made TiO_2_^37^ nanoparticles and are in line with that expected for thermophoretically-driven deposition^38^. The increase in surface roughness with increasing thickness is in good agreement with recent models of the self-assembly of nanoparticles from the gas phase onto flat substrates^39^. The latter predicts that in the diffusion regime the density of first nanoparticle layers are up to 5 times higher than that obtained far from the surface where micro-scale branch-like agglomerated are formed^39^.

**Figure 5:**
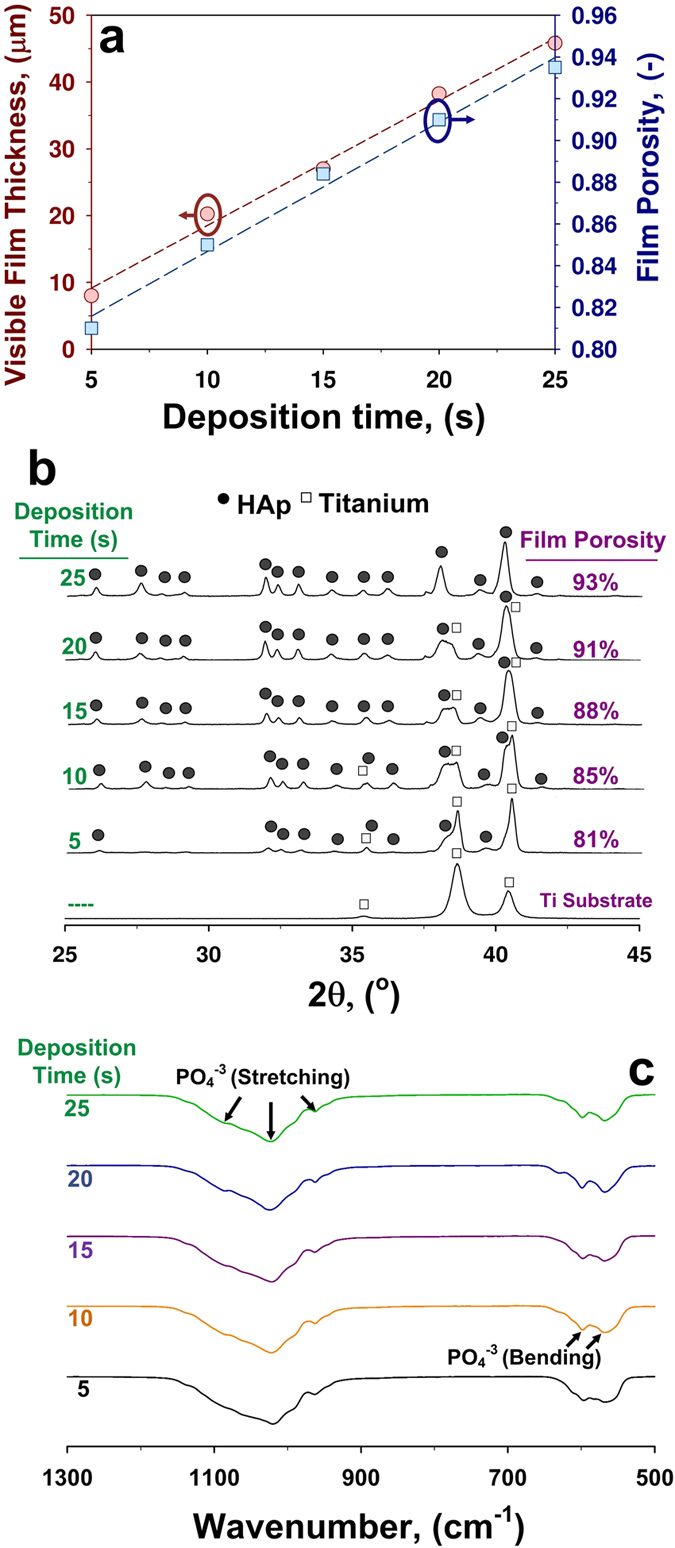
(**a**) Visible film thickness and porosity, (**b**) XRD patterns and (**c**) FTIR spectra of the HAp coatings as a function of the aerosol-deposition time.

The film porosity was computed from the SEM cross-sectional thickness and deposited film mass density (Fig. 5a). The latter increased from 0.54 to 0.99 mg cm^−2^ with increasing deposition time from 5 to 25 s. It is important to notice that during the first 10 s the surface of the substrate heats rapidly from room temperature to ca. 1000 °C. This decreases the thermophoretically driven particle flux from 0.1077 mg cm^−2 ^s^−1^ upon 5 s to 0.0083 mg cm^−2 ^s^−1^ upon 15 s. The latter deposition rate remained constant with no variations up to the longest deposition time (25 s) investigated here. In contrast to previous studies^18^,^25^, the porosity increased from 81% to 93% with increasing deposition time from 5 to 25 s (Fig. 5a). These porosities are significantly lower than that previously achieved by low (98%)^18^,^40^ and moderate temperature (95%)^25^ deposition of flame-made metal-oxide nanoparticles. This is attributed to the low thermodynamic stability of the core-shell nanoparticles undergoing *in-situ* sintering on the substrates during deposition from the gas-phase.

The UNN self-assembly dynamics was investigated as a function of the deposition time by TEM (Fig. 4d,e), XRD (Fig. 5b) and FTIR analysis (Fig. 5c). All the as-prepared coatings had a pure HAp composition (Fig. 5b,c) with no trace of CaO and/or other amorphous impurities. The relative intensity of the HAp peaks increased rapidly with increasing time in line with the rapid increase in film mass density. The HAp crystal size increased from 40 to 67 nm with increasing deposition time from 5 to 25 s. This is up to 4 times larger than the crystal size of the particles collected from the aerosols at the same atomization pressure (Fig. 2c). It confirms that significant densification of the coatings is due to *in-situ* sintering during deposition. The higher porosity of the thicker films is attributed to the self-assembly dynamics leading both to less particle neighbours and larger inter-branch pore as the film thickness increases^39^.

TEM analysis of the HAp particles collected from the substrates (Fig. 4e) revealed that all the core-shell structure had undergone a phase transformation resulting in a homogeneous HAp composition similar to that obtained upon sintering at 800 °C. The purity of the as-prepared coatings was assessed by FTIR analysis (Fig. 5c, Figure S4) revealing a pure inorganic composition. The FTIR main peaks at 550–606 cm^−1^ and 960–1080 cm^−1^ were attributed to the phosphate bending and stretching, respectively. This is in line with previous studies and further confirms the achievement of a highly pure HAp phase. This is particularly important as the presence of CaO in HAp ceramics, designed for medical applications, is not desirable due to the conversion of CaO into Ca(OH)_2_^33^. This results in gradual tension and formation of hairline cracks in the ceramic material^41^ undermining its mechanical properties.

### Cellular adhesion and Infiltration

**Figure 6:**
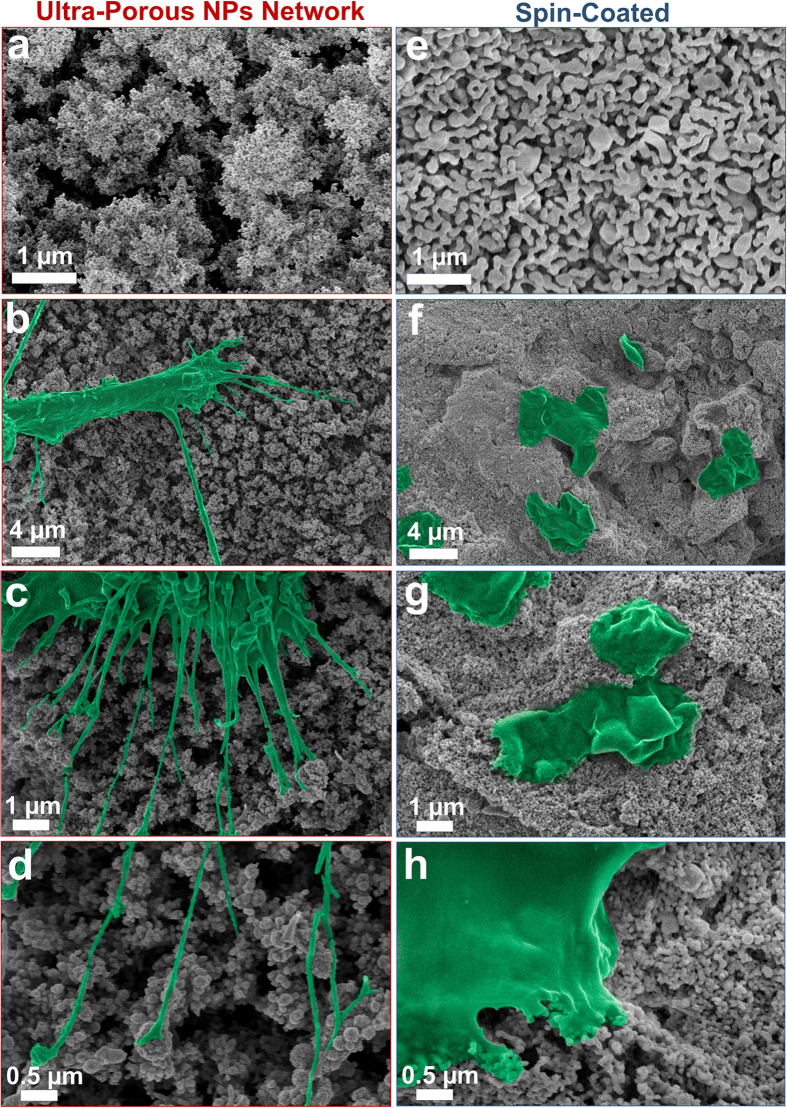
Representative SEM images of (**a**) the flame-made ultra-porous nanoparticle networks (UNN) and (**b**) a dense spin-coated surface. False colored SEM micrographs of the osteoblast growth after 14 days on the surface of the (**b**–**d**) UNN and (**f**–**h**) dense spin-coated surfaces. On the ultra-porous nanoparticle network surface, the cell-cell and cell-materials interactions resulted in a visible attachment of osteoblasts to the coating surface (**b**,**c**). Magnifications show (**d**) the formation of nano-scale filopodia. In stark contrast, no cellular infiltration and filopodia formation was observed on the dense spin-coated surfaces (**f**–**h**).

Promoting **c**ellular adhesion and infiltration into the ultra-porous HAp coatings are recognised as the first key steps for improving the osseointegration on metallic implants. Here, the biological performance of these three-dimensional UNN morphologies was explored using primary Swiss mice osteoblasts as a representative system for the interface of bone implants. To this aim, murine femur cells with osteocalcin producing osteoblasts were cultured in triplicate on HAp coated and spin coated Titanium alloys. Figure 6 shows magnifications of SEM micrographs depicting cell-cell and cell-coating interactions after 14 days on a UNN and a spin-coated surface. The osteoblast were firmly attached to the ultra-porous coating (Fig. 6b) and responded to its morphology by formation of nano-scale filopodia of less than 100 nm in diameter (Fig. 6c,d). Filopodia are cytoskeletal projections of the cell membrane that probe the microenvironment and are known as a mode of recognition and interaction for the cells^42^. The latter infiltrated the coatings through its macro- and nano-pores. Here, a unique extensive penetration of nano-scale filopodia and cell infiltration into the inorganic coatings was observed. Previous studies^43^,^44^ have reported that highly branched cellular morphologies on Ti implant surfaces resulted in higher mineralization with no significant differences in cell viability when compared with control substrate. Thus, this cellular response to our UNN indicates that our structures have higher osseointegration and in turn higher osteoconductivity than current plasma spraying technologies.

In stark contrast, the dense morphology obtained by spin-coating of the same flame-made nanoparticles resulted in poor cell adhesion and infiltration. These spin-coated surfaces were characterized by a smoother morphology having similar nano-scale texture (Fig. 6e and Figure S5b) to the UNN coatings (Fig. 6a and Figure S5a) but lacking the hierarchical micro-structure of the latter (Fig. 6a). SEM analysis of these spin-coated samples revealed that most of the cells failed to adhere to the nano-textured surface (Fig. 6f–h). Furthermore, no cellular infiltration and filopodia formation was observed. This cellular response is similar to that obtained with other approaches such as plasma spray^45^,^46^, sol-gel^47^ and magnetron sputtering^45^ that have been extensively used to fabricate nanostructured HAp surfaces^47^. These dense morphologies have not resulted in cellular infiltration inside the coating structure and only seldom in micro-scale filopodia formation. This is attributed to their micro-scale flat surface that do not may also result in poor cell adhesion^48^.

**Figure 7:**
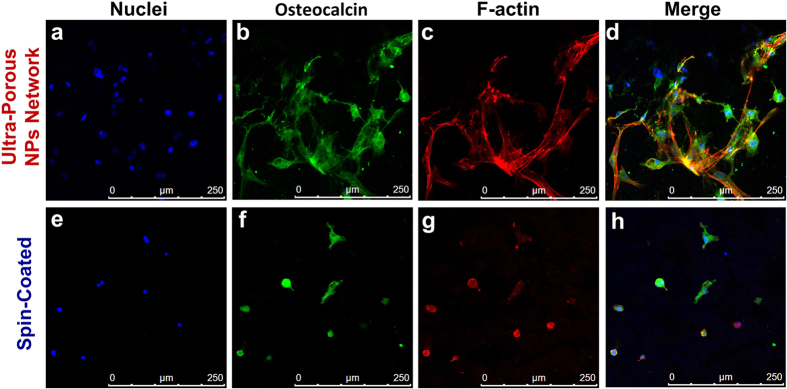
Immunostaining images of (**a**,**e**) nuclei, (**b**,**f**) osteocalcin and (**c**,**g**) F-actin proteins on the surface of (**a**–**d**) the ultra-porous nanoparticle network with an aerosol-deposition time of 20 s and a (**e**–**h**) spin-coated sample. Topographical merges of the nuclei, F-actin and osteocalcin distribution on (**d**) flame-made and (**h**) spin-coated samples.

The very positive response of the osteoblasts to the UNN morphology was confirmed by immunostaining analysis of two key cytoskeletal markers, F-Actin (Fig. 7a) and Osteocalcin (Fig. 7b). Osteocalcin is a non-collagenous protein that is known to be secreted only by functional bone forming cells, osteoblasts^49^. F-Actin is a linear polymer microfilament that is essential for cellular functions such as mobility and contraction during division. The expression of cytoplasmic F-Actin and secreted osteocalcin (Fig. 7c) by bone cells on these ultra-porous and micro-rough morphologies suggest that its three-dimensional structure and composition positively influence the key cellular processes such as adhesion and protein expression. Both are required for osteoconduction and long-term osseointegration of bone implants. In natural bone, the structure of the extracellular matrix and function of cells are regulated by macro- and nano-scale cues provided by both the stroma and parenchyma. These include a pure composition, micro- and nano-scale pore sizes and, in the most active regions of the bone, up to 93% porosity that are all well matched by these flame-made structures. The pore size distribution of the flame-made coatings was investigated by white light interferometry (WLI) and shows that the pore size reaches up to hundreds of micrometer (Figure S6a). Although this is still lower than that of cancellous bone, it is a significant improvement over plasma-sprayed coatings and spin-coating (Figure S6b), where the coatings feature flat surfaces or sub-micrometer pore size distributions. These promising results substantiated by the excellent morphological response of the osteoblasts suggest that the UNN morphology is highly desirable for cellular growth^50^,^51^.

In summary, we have presented a biomimetic, fully inorganic material structure for tissue engineering that can be ultilized to study the early events of osseointegration and osteoconduction. Ultra-porous nanoparticle networks of nanocrystalline hydroxyapatite with up to 93% porosity were fabricated by a scalable and low-cost approach that enables the ultra-rapid coating of commercial grade implants. The response of bone cells to these morphologies was assessed by primary osteoblast cultures. It was found that the nano- and micro-scale hierarchical structures of the UNN surface demonstrated superior cell infiltration and nanoscale cell-cell and cell-biomaterial interaction that can result in enhanced osseointegration. These promising results demonstrate the potential of this scalable synthetic platform and structural design for the fabrication of biomaterials for bone tissue engineering with application extending from osseointegration of implantable devices to regenerative medicine.

## Methods

### Precursor Preparation

Flame spray pyrolysis (FSP) was used for the synthesis and direct deposition of hydroxyapatite (HAp) nanoparticles. Nanoparticles were prepared as follows: calcium naphthenate (Sigma Aldrich, 2(C_11_H_7_O_2_)Ca, ca. 35% in mineral spirits (4% Ca)) and tributyl phosphate (Sigma Aldrich, C_12_H_27_O_4_P) were mixed together at a Ca/P molar ratio of 1.667, matching the natural HAp composition. The solution was supplied at a rate of 5 mL min^−1^ through a needle and dispersed into a fine spray with an oxygen dispersion of 5 L min^−1^. This spray was ignited by a supporting annular premixed methane/oxygen flame (CH_4_ = 1.2 L min^−1^, O_2_ = 3.2 L min^−1^).

### HAp Coating Synthesis

Nanoparticles were deposited by orthogonal impingement of hot aerosol generated by FSP on the substrate placed at 6 cm height above the burner (HAB). The substrates were 1 mm thick Titanium alloy (Ti-6Al-4V, grade 5, Revolution Advanced Metals & Materials) disks with a diameter of 15 mm. Before nanoparticles deposition, the disk surface was cleaned in an ultrasonic bath with ethanol for 20 min. The aerosol temperature (T_F_) on the exposed substrate surface was measured by digital thermometer (RS, model #206-3738). Dense morphologies were obtained by spin-coating of the same flame-made HAp nanoparticles collected on glass-fiber filters placed at 50 cm HAB. A quantity of 1 mL pure water and acetyl acetone with volume ratio of 10:1 was added to the 0.3 g of HAp particles with 2 droplets of Triton X-100 as a surfactant. A spin coater (VTC-100 Vacuum Spin Coater, MTI Corporation) was used at a speed of 1000 rpm for 60 s to spin-coat the above solution on the Ti disk. These films were sintered at 850 °C for 1 h to remove all impurities and achieve a 100% pure HAp. The morphology and patterning characteristics of the deposited particles and films were investigated by a Hitachi H7100FA transmission electron microscope (TEM) at 100 kV and an analytical scanning electron microscopy (Zeiss Ultraplus FESEM) at 3 kV. The crystal phases, size (d_XRD_) and surface compositions were analyzed by X-ray diffraction with a D_2_ Phaser (Bruker, U.S.A) and Fourier transform infrared spectroscopy (FTIR-ATR, Bruker-Alpha, U.S.A). The average crystal sizes of the HAp and calcium oxide phases were computed form the XRD peaks in the 2θ range of 30–35° and 35–40°, respectively. The HAp specific surface area (SSA) was measured by N_2_ adsorption/desorption at −196.15 °C (Micromeritics, TriStar II, U.S.A), after degassing at 300 °C for 4 h. The HAp/Ti alloy interface strength was investigated under the ASTM D4060-14 standard with a Taber abrasion (Dongguan Jianqiao Testing Equipment Co., LTD, Model JQ-802A). The test was performed under two-body (high-stress) condition using a pair of abrading wheels 52 mm in dimeter at 250 g load. The morphology of all coatings was investigated with a Zeiss Ultraplus FE-SEM at 3 kV and white light interferometer (WLI) (Veeco, Wyko NT9100, U.S.A). High temperature differential scanning calorimetry (DSC) analysis was conducted from 30 to 1000 °C at a 10 °C min^−1^ ramp under nitrogen atmosphere in a STA 8000 (Perkin Elmer, USA).

### Cell Culture

This procedure was approved by the Animal Research Ethics Committee of Australian National University (Reference Nr. A2013/41). Primary osteoblasts were isolated from femur bone of adult Swiss mice according to the ANU animal ethics guidelines. The femur was cleaned thoroughly, minced and digested using collagenase (Sigma Aldrich) at a concentration of 10 mg mL^−1^ at 37 °C for 20 min. The digested mass was washed with phosphate buffered saline (PBS) and plated on tissue culture plates (TCP) and maintained with Dulbecco’s Modified Eagle’s medium (DMEM - HyClone) containing 10% foetal bovine serum (FBS, Gibco) and 1% penicillin streptomycin antibiotics (HyClone). Confluent cultures were mechanically detached using cell scrapers (HyClone) and seeded at a density of 10,000 cells per sample (HAp coating on Ti substrate) in 24 well plates.

### Biological SEM sample preparation

Cell morphology was assessed after 14 days of culture. Culture medium was replaced with 3% glutaraldehyde and incubated for 2 h at room temperature for cell fixation. After fixation, the wells were washed with deionized water. The samples were then dehydrated in a series of graded ethanol solutions with final dehydration in 100% ethanol. The samples were then treated with hexamethyldisilazane (HMDS) and allowed to air dry.

### Immunofluorescence analysis

Isolated primary murine bone cells cultures on the HAp coated samples were washed with PBS, and fixed with 4% paraformaldehyde (Sigma-Aldrich) for 10 min. Cells were permeabilized with 0.1% Triton X-100 (Sigma-Aldrich), blocked for non-specific staining using 3% bovine serum albumin (BSA, Sigma) and then immunostained with Phalloidin-TRITC (Millipore) and anti-osteocalcin antibody (R&D systems) for 1 h at room temperature. Thereafter, they were revealed with anti-species-specific Alexa fluor 488 (Life Technologies). The samples were mounted using anti-quenching mounting medium (Vectashiled, Vector laboratories) and observed under a fluorescence microscope for imaging (Olympus IX 71).

## Additional Information

**How to cite this article**: Nasiri, N. *et al.* Ultra-Porous Nanoparticle Networks: A Biomimetic Coating Morphology for Enhanced Cellular Response and Infiltration. *Sci. Rep.* **6**, 24305; doi: 10.1038/srep24305 (2016).

## Supplementary Material

Supplementary Information
