# Supplementary material for: Ultra-Porous Nanoparticle Networks: A Biomimetic Coating Morphology for Enhanced Cellular Response and Infiltration
Source: Sci Rep. 2016 Apr 14;6:24305. doi: 10.1038/srep24305 (PMC4830967; doi:10.1038/srep24305)
Supplement: Supplementary Information [file srep24305-s1.pdf]

Supplementary Information to

Ultra-Porous Nanoparticle Networks: A Biomimetic Coating Morphology for Enhanced Cellular Response and Infiltration

*Noushin Nasiri[1], Anthony Ceramidas[1], Shayanti Mukherjee[2], Anitha Panneerselvan[2], David R. Nisbet[2] and Antonio Tricoli[1]**

[1]Nanotechnology Research Laboratory, Research School of Engineering, Australian National University, Canberra 2601, Australia
[2]Laboratory of Advanced Biomaterials, Research School of Engineering, Australian National University, Canberra 2601, Australia

*Corresponding author: Antonio Tricoli, PhD
E-mail: antonio.tricoli@anu.edu.au
Tel: +61 2 6125 1696

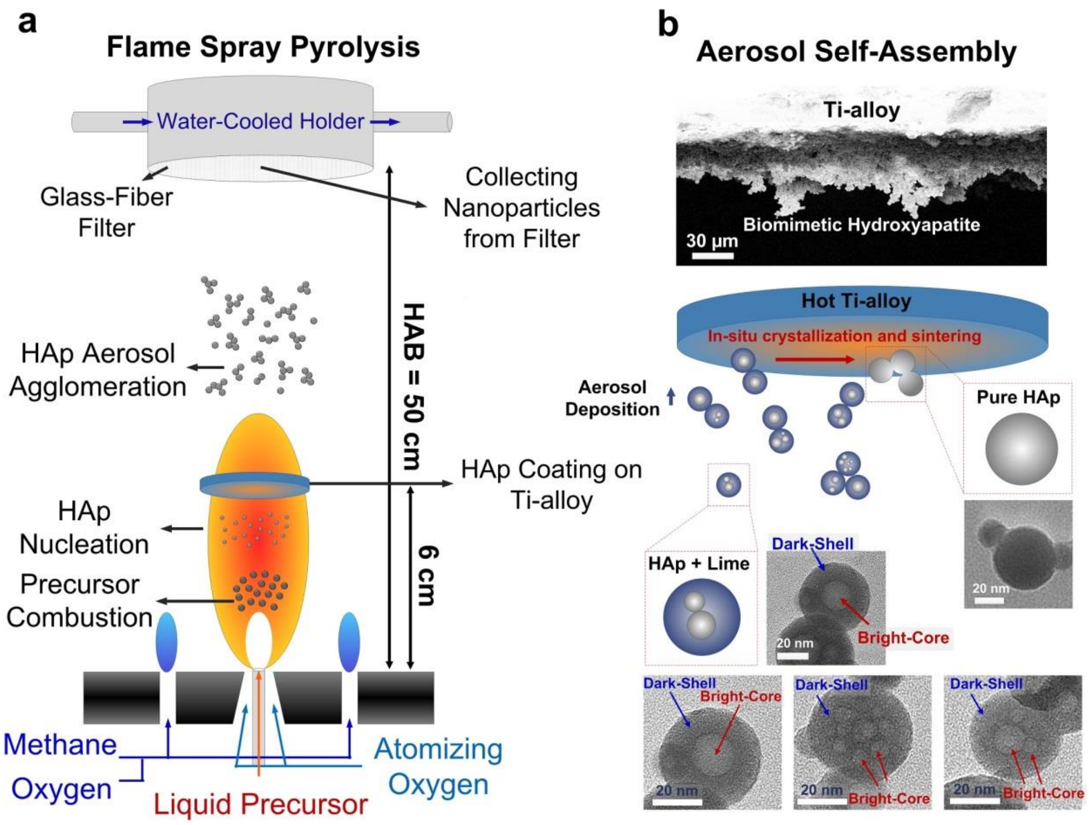

Figure S1. Schematics of (a) flame spray pyrolysis synthesis and (b) aerosol self-assembly of three dimensional ultra-porous films made of HAp nanoparticles at 6 cm height above the burner (HAB). The produced HAp nanoparticles have core-shell structure after nucleation while aerosol deposition on hot Ti-alloy leads to a pure HAp phase with no detectable trace of lime and other impurities.

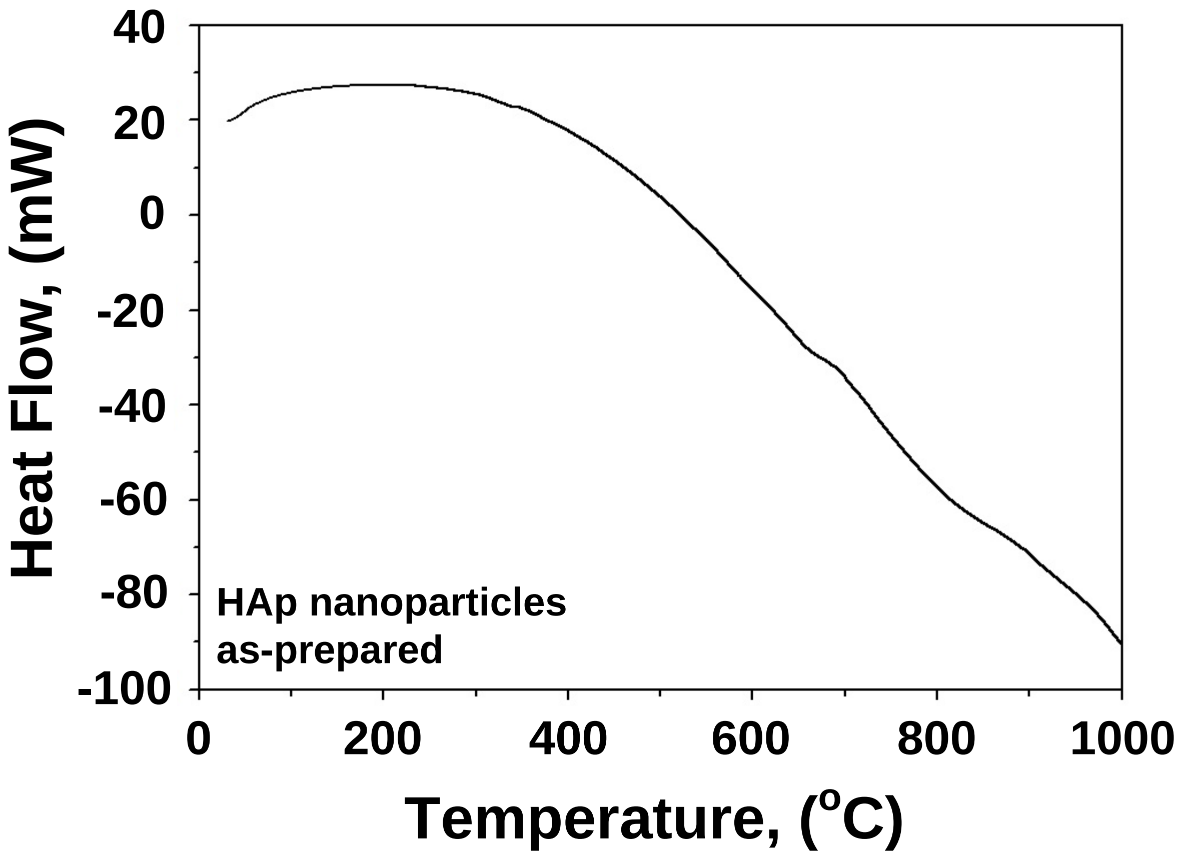

Figure S2. Differential scanning calorimetry (DSC) of the HAp nanoparticles collected from filter at an atomization pressure of 7 bar reveals no crystallization and decomposition up to 1000 ºC.

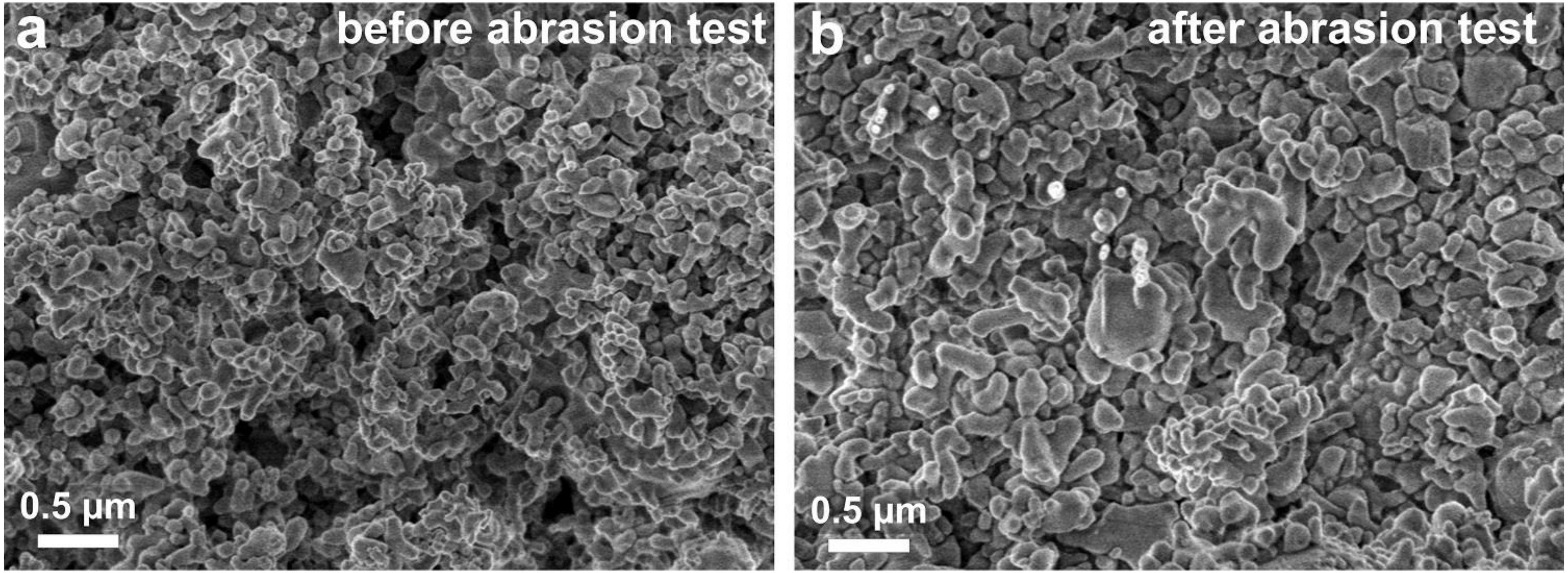

Figure S3. SEM micrographs of HAp UNN morphology (a) before and (b) after 10 cycles abrasion test showing minimal surface restructuring.

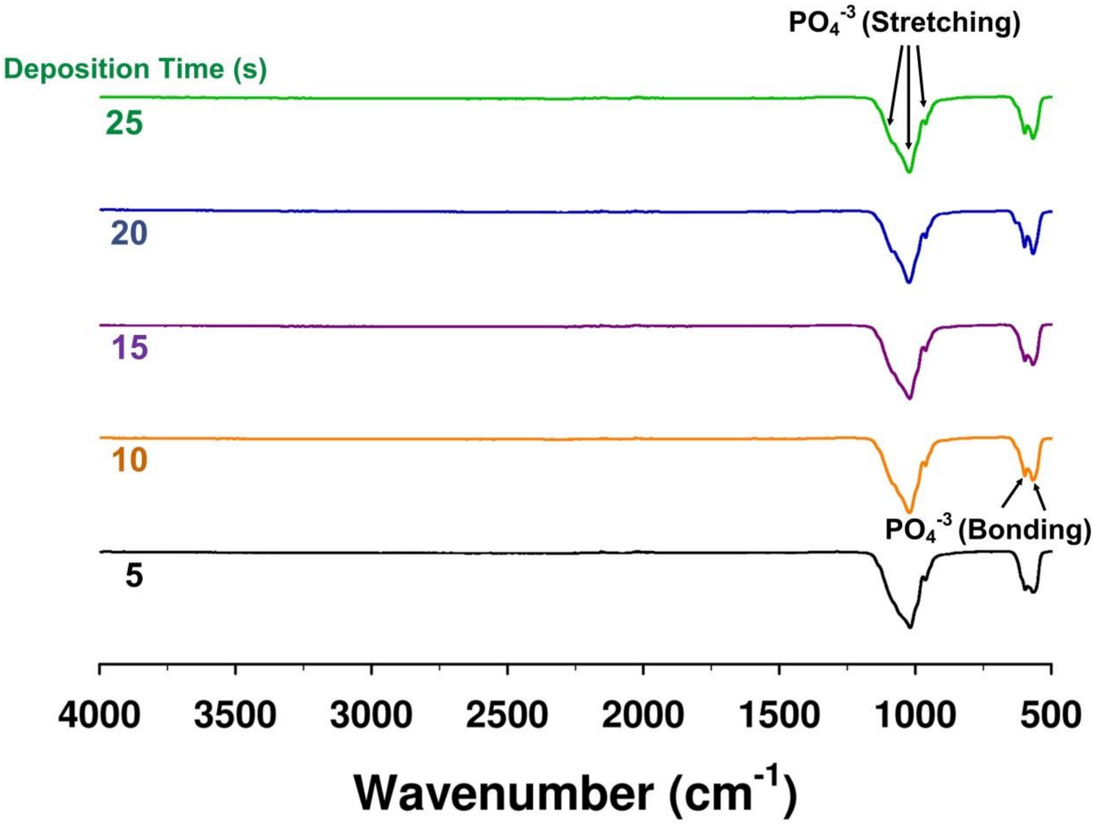

Figure S4. FTIR spectra of the HAp coatings as a function of the aerosol-deposition time.

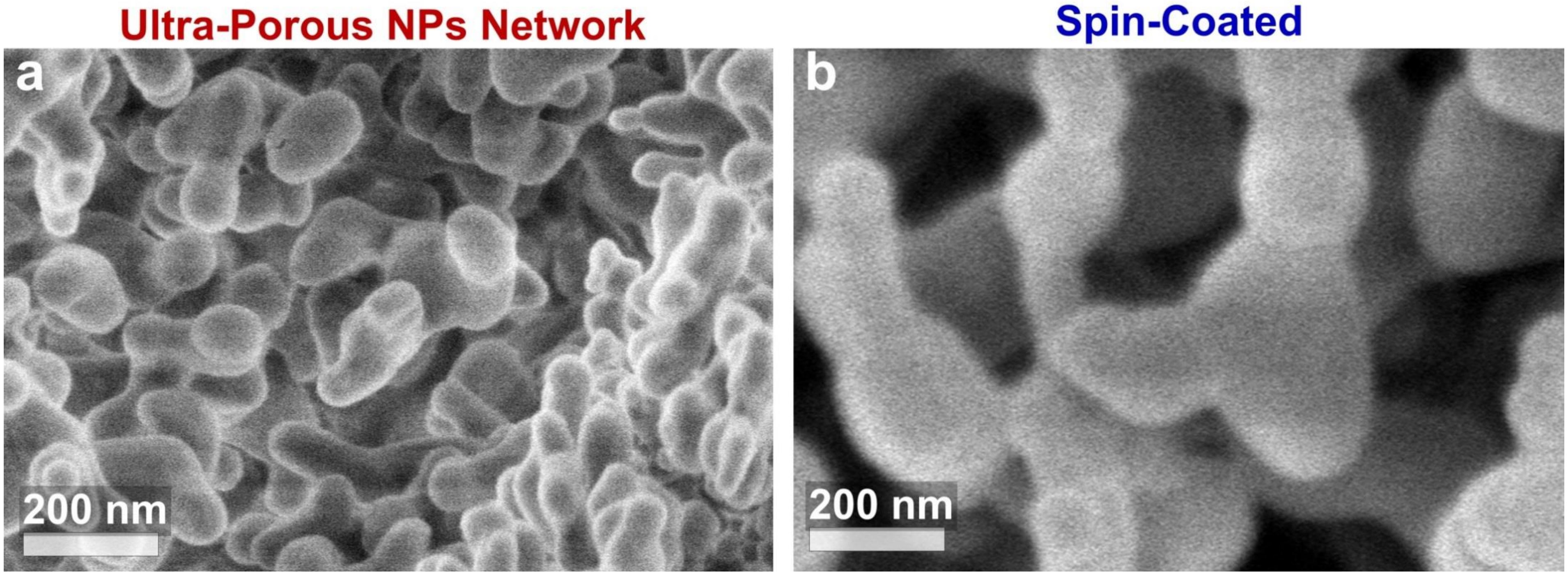

Figure S5. High magnification SEM images of (a) ultra-porous nanoparticles network made by flame spray pyrolysis compared to (b) spin-coated samples composed of the same flame-made particles.

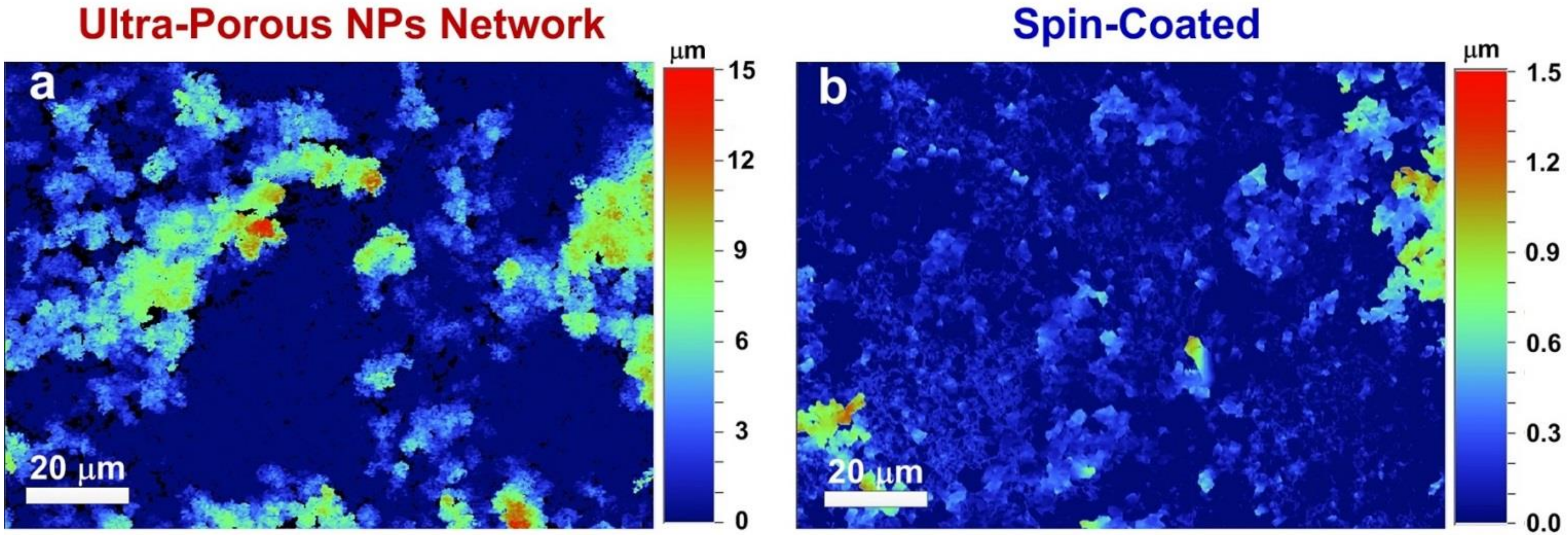

Figure S6. White Light Interferometry (WLI) micrographs of (a) ultra-porous HAp coating with 25 seconds deposition time compared to (b) spin-coated films.
